# Nutritional Approach in Pediatric Patients with Inflammatory Bowel Disease: Treatment, Risk and Challenges

**DOI:** 10.3390/nu17223545

**Published:** 2025-11-13

**Authors:** Maria Elena Capra, Arianna Maria Bellani, Martina Berzieri, Anna Giuseppina Montani, Tullia Sguerso, Valentina Aliverti, Gianlorenzo Pisseri, Susanna Esposito, Giacomo Biasucci

**Affiliations:** 1 Pediatrics and Neonatology Unit, Guglielmo da Saliceto Hospital, 29121 Piacenza, Italy; m.capra@ausl.pc.it (M.E.C.); giacomo.biasucci@unipr.it (G.B.); 2 Pediatric Clinic, Azienda Ospedaliero-Universitaria, 43126 Parma, Italy; ariannamaria.bellani@unipr.it (A.M.B.); martina.berzieri@unipr.it (M.B.); annagiuseppina.montani@unipr.it (A.G.M.); tullia.sguerso@unipr.it (T.S.); valentina.aliverti@unipr.it (V.A.); gianlorenzo.pisseri@unipr.it (G.P.); 3 Department of Medicine and Surgery, University of Parma, 43126 Parma, Italy

**Keywords:** Crohn’s disease, ulcerative colitis, children, adolescents, dietary intervention, future perspectives

## Abstract

Inflammatory Bowel Diseases (IBDs), including Crohn’s disease (CD) and ulcerative colitis (UC), have become a growing global health concern in children and adolescents. Pediatric-onset IBD presents unique challenges compared with adult-onset forms, including more extensive disease, impaired growth, delayed puberty, and psychosocial difficulties. While biologic and targeted therapies have advanced disease control, nutritional interventions remain a central component of management. Exclusive enteral nutrition (EEN) is recognized as the first-line therapy for inducing remission in pediatric CD, offering comparable efficacy to corticosteroids with additional benefits for mucosal healing, nutritional status, and growth. Modified dietary approaches, such as partial enteral nutrition and the Crohn’s Disease Exclusion Diet (CDED), show promise for improving adherence and maintaining remission. However, dietary restrictions may lead to deficiencies and psychosocial stress, underscoring the importance of individualized, dietitian-supervised care. The role of nutrition in UC is less defined, but balanced, anti-inflammatory dietary patterns appear beneficial. This narrative review summarizes current evidence on nutritional strategies in pediatric IBD, highlighting their therapeutic potential, limitations, and integration with pharmacologic treatment within a multidisciplinary framework aimed at optimizing outcomes and quality of life.

## 1. Introduction

Inflammatory Bowel Diseases (IBDs), encompassing Crohn’s disease (CD) and ulcerative colitis (UC), have emerged as a significant global health concern in pediatric subjects. Pediatric-onset IBD (PIBD) exhibit distinct clinical, pathophysiological, and prognostic features compared to adult-onset forms. In younger patients, diagnosis is often established at an earlier stage of disease progression, frequently before the onset of related complications. Nevertheless, the identification of IBD during childhood introduces unique challenges, including the psychological burden of living with a chronic condition—affecting both the child and their family—as well as issues such as school absenteeism and impaired linear growth [1].

Over recent decades, the incidence and prevalence of PIBD have increased markedly worldwide, particularly in industrialized countries, suggesting an interplay between genetic predisposition and environmental factors [2,3,4]. These environmental determinants include dietary habits, early-life antibiotic exposure, and urbanization, all of which can influence gut microbiota composition and immune regulation [5,6]. The pathogenesis of IBD is multifactorial, involving genetic susceptibility, intestinal dysbiosis, and immune dysregulation triggered by environmental stimuli. In this context, lack of exercise is an important factor to be considered [7,8].

Pediatric IBD poses a unique clinical challenge due to its aggressive course, impact on growth and pubertal development, and the necessity for long-term treatment. Beyond intestinal symptoms, children often experience nutritional deficiencies, delayed puberty, reduced bone mineral density, and psychosocial difficulties that affect quality of life [9,10]. Consequently, comprehensive management must include not only pharmacologic interventions but also nutritional, psychological, and social support.

Although the introduction of biologic and targeted therapies has improved disease outcomes, nutrition remains a cornerstone in the management of pediatric IBD. Exclusive enteral nutrition (EEN) is now recognized as the first-line induction therapy for pediatric CD, demonstrating efficacy comparable to corticosteroids while promoting mucosal healing, improving nutritional status, and supporting linear growth [8]. In addition, partial enteral nutrition (PEN) and exclusion diets such as the Crohn’s Disease Exclusion Diet (CDED) have emerged as promising strategies for maintaining remission and enhancing adherence.

The aim of this narrative review is to analyze the nutritional treatment of children and adolescents with IBD, with particular attention to its therapeutic potential, associated risks, and implementation challenges within a multidisciplinary framework designed to optimize clinical and developmental outcomes.

## 2. Methods

The objective of this narrative review was to analyze nutritional treatment strategies and their associated risks and challenges in children and adolescents with IBD. The review was conducted in three main stages: (1) literature search, (2) screening of abstracts and full texts, and (3) synthesis and evaluation of results. Relevant studies published between 1990 and June 2025 were retrieved from the following electronic databases: PubMed, EMBASE, Scopus, ScienceDirect, Web of Science, and Google Scholar. The search strategy combined the following keywords and Boolean operators: “inflammatory bowel disease” OR “Crohn’s disease” OR “ulcerative colitis” AND “diagnosis” OR “treatment” OR “weight modifications” OR “nutrition” OR “enteral” OR “nutritional challenges” OR “eating behavior.”

Eligible publications included double-blind randomized controlled trials, controlled clinical trials, randomized placebo-controlled studies, and systematic reviews conducted in pediatric populations. The search was restricted to English-language articles with available full texts. To enhance completeness, the reference lists of selected papers were manually screened to identify additional relevant studies.

Following the search, duplicate records were removed, and all remaining abstracts were independently reviewed to ensure relevance to the topic. Studies meeting inclusion criteria were then assessed in full to extract data on nutritional interventions, outcomes, and related risks. Discrepancies during the selection process were resolved by discussion among the authors until consensus was reached.

Given the heterogeneity of study designs and outcomes, a narrative synthesis was employed instead of a meta-analysis. The collected evidence was organized thematically to address the epidemiology, pathophysiology, diagnostic aspects, pharmacological and nutritional treatments, and nutritional risks associated with pediatric-onset IBD.

## 3. Inflammatory Bowel Disease

### 3.1. Epidemiology

The incidence of IBD has risen substantially over recent decades, particularly in industrialized regions such as Northern Europe and North America, where the highest global rates are reported. In contrast, Southern Europe, Asia, and the Middle East continue to display lower rates, although these areas are also witnessing steady increases [2]. Recent evidence confirms a global surge in PIBD, with a growing number of cases diagnosed among children and adolescents [3].

Data from the Global Burden of Disease Study (1990–2019) indicate a worldwide rise in PIBD incidence, with the greatest burden observed in high Socio-Demographic Index (SDI) countries. In 2019, incidence reached 6.3 per 100,000 person-years in these regions, with Canada, Denmark, and Hungary reporting the highest rates. Conversely, countries with lower SDI values recorded greater mortality and disability-adjusted life years (DALYs) linked to PIBD [4].

A large European review spanning 51 countries (1970–2018) confirmed marked increases in PIBD, especially in Northern Europe, with pediatric CD incidence reaching 9–10 per 100,000 population [5]. Italian registry data from 2009–2018 reported lower rates (1.59–2.04 per 100,000 in individuals < 18 years), with UC slightly more common than CD [6].

Globally, IBD incidence has plateaued in older children, yet an upward trend persists among those under five years of age. Incidence appears slightly higher in boys (1.0 per 100,000) compared with girls (0.91 per 100,000) [4]. Despite substantial progress in epidemiological mapping, population-based pediatric studies remain limited, and age-specific trends vary across cohorts. Factors contributing to apparent incidence shifts include increasing Westernization of lifestyle, improved awareness that IBD can present in very young children, and enhanced diagnostic access through pediatric gastroenterology networks, endoscopy, and imaging [7].

### 3.2. Chron’s Disease and Ulcerative Colitis

IBD encompasses chronic, immune-mediated inflammatory disorders of the gastrointestinal (GI) tract. Although the precise pathogenesis remains elusive, it is widely accepted that IBD arises in genetically susceptible hosts following environmental or microbial triggers that disrupt gut homeostasis, resulting in dysbiosis and aberrant immune activation [8]. The main subtypes—CD and UC—are complemented by a less common indeterminate form termed IBD-unclassified (IBD-U) [9].

CD and UC share immunoinflammatory features but differ in distribution and histopathology. CD can involve any segment from mouth to anus, with discontinuous, transmural inflammation that may lead to strictures, fistulas, or abscesses. UC, in contrast, is confined to the colon and rectum, showing continuous mucosal inflammation extending proximally from the rectum; granulomas, when present, are diagnostic of CD [10,11].

A subset of patients—most often children with UC—develop primary sclerosing cholangitis (PSC), forming a distinct phenotype known as PSC-associated IBD (PSC-IBD). This condition typically shows proximal colonic inflammation with relative rectal sparing and “backwash” ileitis, while colitis symptoms may be milder than in classic UC. Importantly, PSC-IBD confers a markedly elevated risk for cholangiocarcinoma and colorectal cancer [12].

Monogenic IBD, although rare, is more frequent among very-early-onset cases (VEO-IBD, <6 years), where 13–41% of infants may harbor single-gene mutations. These include primary immunodeficiencies and epithelial barrier disorders requiring tailored therapeutic approaches distinct from conventional IBD treatment [13].

The pathogenesis of both diseases reflects a convergence of genetic, microbial, and immune factors [8,14]. While traditionally considered distinct, CD and UC share overlapping immunogenetic and molecular pathways. Genome-wide association studies have identified common loci—such as NOD2, IL23R, and ATG16L1—suggesting shared mechanisms and raising the hypothesis that both may represent ends of a single inflammatory spectrum [15].

Microbiome studies also demonstrate distinct yet overlapping dysbiotic patterns in CD and UC [16]. In children, clinical overlap can obscure diagnosis, necessitating classification as IBD-U until phenotype evolution allows redefinition [17]. These challenges underscore the need for integrative diagnostic models combining clinical, histologic, microbial, and molecular markers to enhance precision and guide individualized care [18].

### 3.3. Diagnostic Criteria

PIBD frequently presents atypically compared with adult-onset disease. Up to one-fifth of children initially display extraintestinal signs—such as growth failure, anemia, or perianal disease—before gastrointestinal symptoms appear, contributing to diagnostic delays [17].

Diagnosis is established by confirming intestinal inflammation and excluding mimicking conditions through clinical evaluation, endoscopy, histopathology, and imaging [19]. The Paris Classification has standardized pediatric phenotypes and improved diagnostic consistency [20]. Recent advances such as magnetic resonance enterography (MRE), capsule endoscopy, and fecal or serological biomarkers have enhanced diagnostic accuracy while minimizing radiation exposure [21].

Bloody diarrhea predominates in UC, whereas CD often manifests with abdominal pain, weight loss, fever, or growth retardation—though the full triad appears in only a quarter of patients [22]. Extraintestinal manifestations occur in 6–23% of pediatric cases, particularly in older children [23].

Endoscopic and histologic evaluation remain central: UC shows continuous mucosal inflammation beginning at the rectum, whereas CD features segmental, transmural lesions with aphthous ulcers or cobblestoning. Noncaseating granulomas are highly suggestive of CD [24,25]. IBD-U applies when findings are indeterminate despite complete workup [26].

Serological markers, including ASCA and pANCA, support differential diagnosis but lack standalone diagnostic accuracy [27]. Laboratory tests often reveal anemia, thrombocytosis, or raised CRP/ESR, though normal results do not exclude disease [28].

Fecal calprotectin (FC) is the most reliable noninvasive marker for intestinal inflammation (AUC 0.93 for IBD diagnosis) [29], though elevated levels are not specific and may occur in infections or celiac disease [30]. FC is especially valuable for triaging children with nonspecific symptoms [31].

Screening for hepatobiliary and ocular comorbidities is recommended, while MRE remains the imaging modality of choice for small-bowel assessment [32]. Wireless capsule endoscopy and abdominal ultrasonography may complement evaluation in selected cases [33].

In the diagnostic process, a diagnostic questionnaire, such as the Harvey–Bradshaw index (HBI), may be a useful tool. The five domains that make up the HBI are abdominal mass, abdominal discomfort, complications, overall health, and the quantity of liquid or extremely soft stools. The total HBI score was determined by adding the scores from each of the five domains. Depending on how many stools a patient reports having each day, the overall HBI score might vary from 0 to >16. Conventionally, remission is indicated by a total HBI score of less than 5, mild severity is indicated by a score of 5–7, moderate severity is indicated by a score of 8–16, and severe disease is indicated by a score of ≥16 [34].

### 3.4. Immunopathogenesis

Rising IBD incidence has been linked to modern environmental and lifestyle changes influencing immune regulation. Key contributors include altered gut microbiota, antibiotic exposure, Westernized diets, smoking, and low vitamin D levels. Early-life antibiotic use and infectious gastroenteritis may disrupt microbial balance and heighten susceptibility. The “hygiene hypothesis” posits that reduced microbial exposure diminishes immune tolerance, predisposing to inflammatory disorders [35]. Reduced microbial diversity and loss of protective taxa—such as Helicobacter pylori—have been correlated with higher rates of immune-mediated diseases. Collectively, these findings support a model in which microbial diversity fosters immune regulation via tolerogenic dendritic and T regulatory cells, whereas reduced diversity triggers proinflammatory immune activation consistent with IBD pathogenesis.

### 3.5. Risk Factors

Environmental influences play a critical role in IBD onset, particularly during early life. A recent umbrella review of 53 meta-analyses identified several exposures linked to disease risk [36]. For CD, smoking, urban residence, cesarean delivery, prior appendectomy or tonsillectomy, antibiotic and oral contraceptive use, and vitamin D deficiency were associated with increased risk. Protective factors included breastfeeding, early microbial exposure (pets or farm environments), and adequate vitamin D [37].

Diet represents one of the most modifiable risk factors. Diets high in processed meats, refined sugars, and saturated fats are associated with greater IBD risk, whereas fiber-rich, plant-based diets confer protection by supporting microbial diversity and anti-inflammatory metabolite production [37,38].

In PIBD, early-life exposures—including delivery mode, antibiotic use, and infant feeding—exert strong influences on immune and microbiome development [39,40,41,42]. Cesarean delivery and antibiotic exposure disrupt early colonization, while breastfeeding supports immune tolerance. Environmental microbial diversity, as proposed by the hygiene hypothesis, may reduce IBD risk [43]. Lifestyle and diet during childhood further modulate risk, with high-fiber diets offering potential protective effects [44,45]. Identification of genetically at-risk children may allow preventive strategies targeting modifiable exposures [37].

### 3.6. The Role of Microbiome

The intestinal microbiota, established early in life, is fundamental to immune and metabolic homeostasis [46]. Colonization begins at birth and evolves under the influence of delivery mode, diet, and environment. Commensal microbes interact with innate immune receptors such as TLRs and NLRs, shaping immune tolerance and mucosal defense [35].

IBD arises from disruption of this host–microbe balance, resulting in aberrant immune responses to commensals [47]. Dysbiosis is characterized by reduced microbial diversity, loss of beneficial species (e.g., *Faecalibacterium prausnitzii*), and expansion of *Proteobacteria* [48,49,50,51,52,53]. These shifts increase mucosal permeability and immune activation [48]. The composition of microbiota in patients with IBD shows significant differences compared to healthy individuals. Beta-diversity, which measures the variation in microbial types across multiple samples, and alpha-diversity, which reflects the variation within a specific ecosystem or sample, are both diminished in the microbiota of IBD patients compared to healthy controls, especially when considering the stability of the microbiome over time [54]. A recent meta-analysis that incorporated 13 studies confirmed that reduced alpha-diversity is a common characteristic of both CD and UC, with a more significant reduction observed in CD (OR of 3.20, 95% CI: 2.09–4.88, *p* < 0.001) [55]. The gut microbiota plays a crucial role in influencing intestinal inflammation, primarily through its interactions with the immune system. The intestinal mucus layer plays a central role in maintaining gut homeostasis. Clarifying the relationship between the gut microbiota and the mucus layer may be crucial to understanding the pathophysiology of IBD. A fundamental aspect of the pathophysiology of IBD is the correlation between compromised barrier function and the composition of the gut microbiome. According to recent data, poor intestinal permeability may be associated with a lower prevalence of *Adlercreutzia*, which has anti-inflammatory qualities, and a higher abundance of *Colidextribacter*, which contributes to cellular oxidative stress. Furthermore, the loss of microbial processes linked to the production of the amino acids glutamate, tryptophan, and threonine raises the possibility that metabolites of the microbiota are responsible for the altered permeability of the gut [56,57,58].

The gut microbiota may cause harm beyond the gut. In actuality, immune cells can be directly stimulated to release a variety of proinflammatory cytokines when infections move from mucosal surfaces into peripheral tissues.

Genetic variants such as *NOD2* and *ATG16L1* influence microbial composition and autophagy pathways, linking host genetics and microbiota to disease [50]. Epigenetic mechanisms, including short-chain fatty acid–mediated modulation of gene expression, are increasingly recognized in mucosal inflammation [52].

Therapeutic strategies targeting the microbiota are emerging. Exclusive enteral nutrition (EEN), a cornerstone of pediatric CD treatment, likely acts through microbiome modulation [27,59]. Although probiotics have shown experimental benefits, clinical evidence remains inconsistent, and current guidelines limit their use to selected UC cases [60]. Fecal microbiota transplantation (FMT) shows potential for restoring microbial balance but requires further validation [49].

### 3.7. Genetic Issues

Genetic predisposition plays a central role in PIBD, with both polygenic and monogenic forms contributing to disease heterogeneity. Although monogenic IBD accounts for <0.5% of pediatric cases, early identification is critical as these patients often exhibit severe, treatment-resistant disease requiring individualized management [19].

Children with very-early-onset IBD (VEO-IBD) are most likely to harbor single-gene defects—over 100 have been identified to date—affecting immune regulation or epithelial barrier integrity [61,62,63]. Variants in IL-10 signaling or XIAP genes exemplify defects that precipitate uncontrolled intestinal inflammation [64,65]. Early genetic screening enables precision therapy, including hematopoietic stem cell transplantation in monogenic immune disorders [63,66]. Genetic diagnostics also aid family counseling and contribute to understanding inheritance patterns and disease mechanisms.

## 4. Pharmacologic Treatment for Pediatric Inflammatory Bowel Diseases

Over recent decades, the pharmacologic management of inflammatory bowel diseases (IBD) has evolved substantially (Table 1). Nevertheless, nutritional therapy continues to represent a fundamental component of pediatric IBD care—not only for symptomatic relief but also as a true disease-modifying intervention. In this review, special focus is placed on the therapeutic role of nutrition as part of an integrated management strategy.

For induction of remission in moderate-to-severe disease, corticosteroids remain the traditional first-line pharmacologic option. Agents such as prednisone and budesonide are effective in rapidly reducing inflammation and controlling symptoms; however, prolonged use in children is strongly discouraged because of well-known adverse effects, including growth impairment, delayed puberty, bone demineralization, and mood or behavioral disturbances. Moreover, corticosteroids fail to achieve sustained mucosal healing, now considered a primary therapeutic target in pediatric IBD [67].

To maintain remission, immunomodulators such as azathioprine, 6-mercaptopurine, and methotrexate are frequently prescribed. Although these drugs can reduce relapse rates, their clinical utility is limited by a delayed onset of action, variable individual response, and the need for regular monitoring to detect hepatotoxicity, leukopenia, and rare but serious complications like lymphoma [65].

Biologic therapies, particularly tumor necrosis factor (TNF) inhibitors including infliximab and adalimumab, have transformed the therapeutic landscape for pediatric IBD. These agents induce rapid clinical improvement and are capable of achieving deep remission, encompassing both mucosal and histologic healing. They are especially valuable in patients who are corticosteroid-dependent or refractory to conventional therapy. Nevertheless, biologic use in children requires careful monitoring due to the potential for immunogenicity, infusion or injection reactions, and uncertainties regarding long-term safety [68].

More recently, targeted biologics such as vedolizumab—a gut-selective anti-integrin—and ustekinumab, which blocks interleukin-12 and interleukin-23 pathways, have been introduced for children who fail or cannot tolerate anti-TNF therapy. While early results are encouraging, pediatric-specific data remain limited [69,70]. Despite these therapeutic advances, approximately one-third of pediatric IBD patients ultimately require surgical intervention, underscoring the ongoing need for safer and more effective long-term management strategies [27].

## 5. Nutritional Treatment for Pediatric Inflammatory Bowel Diseases

Nutrition is integral to PIBD care, yet high-quality, disease-specific dietary guidance remains limited [71]. In the absence of clear recommendations, many patients self-impose exclusion diets, which can precipitate macronutrient and micronutrient deficits and may contribute to disordered eating behaviors [72]. Surveys indicate frequent avoidance of major food groups—grains (29%), milk (28%), vegetables (18%), and fruit (11%)—as a strategy to manage symptoms [73].

In a cohort study of 68 children and adolescents (57 CD, 11 UC), Hartman et al. found substantially lower intakes than recommended for energy (78% of requirements) and several nutrients—carbohydrates (61%), magnesium (67%), vitamin C (34%), and fiber (54%). Participants receiving EN or polymeric formula supplements achieved higher intakes of energy, carbohydrates, minerals, and micronutrients than peers on an unrestricted oral diet alone [74].

Pons et al. compared children with active CD, CD in remission, and healthy controls, showing reduced weight and BMI z-scores during active disease and energy intake below estimates in both active (*p* = 0.001) and remission groups (*p* = 0.03). Iron and calcium intakes were inadequate during activity, and calcium remained suboptimal even in remission. Carbohydrate intake was lower in active CD, while fat intake did not differ among groups [75]. Collectively, PIBD confers a high risk of malnutrition and its sequelae, with amplified consequences in growing children.

### 5.1. Macronutrients and Micronutrients Requirement in Patients with pIBD

Studies of energy needs in pediatric CD are inconsistent [76]. Resting energy expenditure (REE) may be elevated during active inflammation, suggesting a hypermetabolic state [77,78], though some data attribute higher measured expenditure to altered body composition rather than increased basal demand per se [79]. These discrepancies reinforce the need for individualized assessment that considers disease activity and composition changes [80]. Overall, total energy needs are often comparable to healthy peers, but careful monitoring is essential to ensure adequacy [76].

#### 5.1.1. Macronutrient Requirements

Current evidence does not support disease-specific targets for carbohydrate or fat beyond standard pediatric guidance (≈45–60% of energy from carbohydrates; age-appropriate fat ranges) [76]. Children with IBD commonly exhibit reduced lean mass with relative increases in fat mass; active inflammation accelerates protein turnover via intestinal protein loss and catabolism, which may also be potentiated by corticosteroids [78]. Elemental or polymeric formulas can mitigate proteolysis [79]. Practically, protein intake should be increased during active disease to offset losses and support growth, while usual age-appropriate protein recommendations suffice during remission [76].

#### 5.1.2. Micronutrient Requirement

Micronutrient deficits arise from malabsorption, intestinal loss, and insufficient intake [76]. Interpretation of serum markers is complicated by inflammation (e.g., selenium, zinc, and folate tend to decrease, whereas ferritin and copper may rise) [80]. Even in clinical remission and in seemingly well-nourished patients, multiple deficiencies are frequently detected [81,82]. Accordingly, the 2017 ESPEN guidance recommends routine annual screening with targeted replacement as needed [76]. Multivitamins alone may not correct specific long-term deficits, necessitating individualized supplementation plans [83].

### 5.2. Exclusive Enteral Nutrition

Nutritional therapy occupies a unique position in PIBD by simultaneously inducing remission and supporting growth. EEN—a complete formula-only regimen for 6–8 weeks—is an established first-line induction therapy for pediatric CD, demonstrating remission rates comparable to corticosteroids with added benefits for mucosal healing, nutritional repletion, and linear growth [70]. Proposed mechanisms include removal of dietary antigens, modulation of the microbiota, intestinal “rest,” and direct anti-inflammatory effects [70]. Despite strong efficacy, adherence can be challenging; nevertheless, children often achieve better adherence than adults, and partial enteral nutrition combined with exclusion diets can aid maintenance for selected patients [70].

Diet also matters in UC, although evidence is less robust. Balanced, anti-inflammatory patterns emphasizing fiber-rich, minimally processed foods may support mucosal health as an adjunct to pharmacotherapy [84].

EEN can be delivered orally or via tube with similar effectiveness [85]. Formulations include elemental (amino acid), semi-elemental (oligopeptide), and polymeric (whole-protein) diets [86,87]. In children with intact intestinal function, polymeric formulas are preferred for palatability, cost, and nutritional completeness [76,88].

EEN, introduced in the 1970s and adopted broadly in the 1990s, is endorsed by ECCO/ESPGHAN as first-line induction therapy for new-onset, mild-to-moderate pediatric CD, providing 100% of daily caloric needs for 6–8 weeks [27]. Caloric volumes are calculated from estimated requirements and the specific formula’s energy density. Mechanistically, benefits likely reflect a combination of exclusion of proinflammatory dietary components, microbiome shifts, intestinal rest, and anti-inflammatory effects [89].

**Figure 1:**
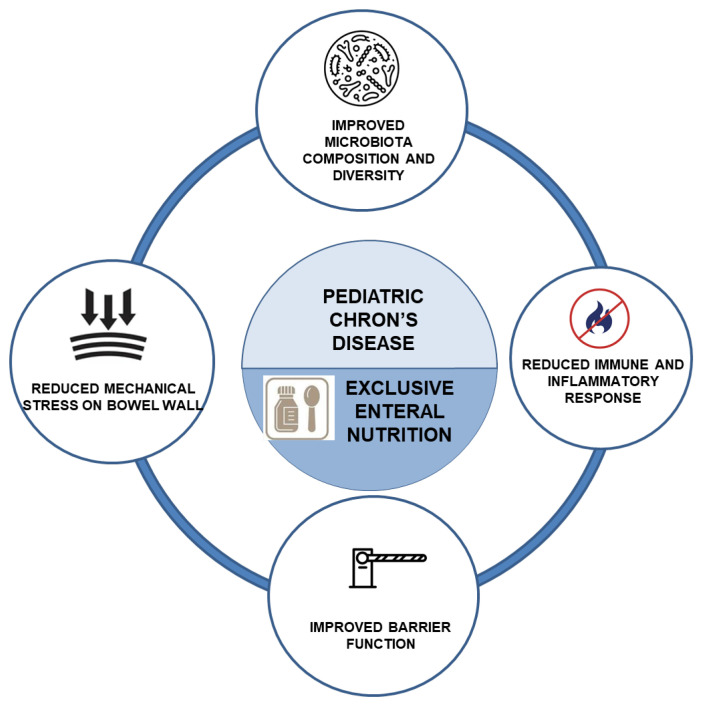
Exclusive enteral nutrition’s (EEN’s) mechanism of action, adapted from [76,85,86,87].

EEN’s mechanism of action is summarized in Figure 1.

Microbiome studies show EEN reduces dysbiosis—lowering Firmicutes (including *Faecalibacterium*), *Bacteroides/Prevotella*, and *Proteobacteriaceae,* with relative increases in *Bacteroidetes* taxa [90,91,92,93]. Some of these gains regress after food reintroduction, with expansion of *Escherichia coli* even during clinical remission [93]. Overall, multiple studies report that EEN achieves remission rates similar to or superior to corticosteroids (Table 2). The studies reported in Table 2 differ from each other in several aspects. The study designs are different. For instance, Connors et al. [94] conducted a retrospective study, whereas Pigneur et al. [95] and Levine et al. [70] conducted a randomized trial. The study populations differ as well: the accepted age range, disease severity, time since last therapy, disease localization, and presence of complications are criteria that vary or are not always clearly reported. Moreover, in some cases, EEN is applied to patients with newly diagnosed CD [70,94,96], while in another study it is not specified or it is also applied to patients with relapses. The main studies considered are also heterogenous in terms of the parameters used to evaluate the effectiveness of EEN (Levine et al. [70] uses CRP as a parameter, Luo et al. [97] and Soo et al. [96] use PCDAI, whereas Pigneur et al. [95] use the Crohn’s Disease Endoscopic Index of Severity). Levine et al. also aim to compare the parameters used for induction of remission.

To date, no robust evidence supports EEN as an effective therapy for active UC [98,99]. One of the persisting challenges in EEN management is the lack of standardized guidance for reintroducing food once exclusive feeding ends. Available studies show no significant difference between early and gradual reintroduction protocols in terms of clinical outcomes [100].

The role of maintenance enteral nutrition (MEN)—the continued use of partial formula intake after induction—remains under investigation. According to ESPEN guidelines, MEN in the form of oral nutritional supplements may improve anthropometric measures and nutritional status and could help extend remission and reduce steroid exposure [101]. Some studies have also explored its potential to prevent postoperative recurrence of Crohn’s disease following intraoperative enteroscopy, although others, such as Gavin et al., did not find significant reductions in relapse rates at 12 months [102].

Despite equivalent remission rates compared with corticosteroids, EEN is generally preferred for children because of its favorable safety profile. Beyond disease control, it can positively affect BMI z-scores and bone turnover markers within three months of initiation, as reported by Werkstetter et al. [103]. Weight-for-age gains have also been documented and appear partially independent of improvements in disease activity.

The main safety concern associated with EEN is vomiting, often linked to the taste or texture of the polymeric formula. Common barriers to adherence include limited flavor options, monotony, and the social challenges of adhering to a liquid-only diet. In a study of children aged 5–17 years with Crohn’s disease, 68% reported inadequate flavor variety, 23% disliked the formula’s taste, and 9% struggled with food cravings. Many described feelings of social exclusion from shared meals and family activities [104].

Up to half of pediatric patients require nasogastric tube administration, and some may refuse EEN altogether for this reason. Adherence can also vary by disease characteristics: in a cohort of 376 children, overall adherence reached 89%, though it was significantly lower in those with colonic involvement or fecal calprotectin levels > 600 μg/g at diagnosis [105]. Other mild adverse effects, including nausea, abdominal pain, flatulence, and diarrhea, are occasionally reported.

In summary, EEN remains the first-line induction therapy for pediatric CD, showing superior safety and comparable efficacy to corticosteroids. However, strong evidence guiding post-EEN food reintroduction is lacking, and further research is needed to define predictors of treatment response—such as disease location or phenotype. Current recommendations do not require phenotype-based adjustments when initiating EEN [106]. In adult patients, food antigens elicited strong immune responses. It may be possible to use the quantity of IgG-positive foods as a CD diagnostic indicator. Maintaining illness remission may be possible with a diet based on food antibody testing, but further studies are needed in this context [107].

### 5.3. Partial Enteral Nutrition and CD Exclusion Diet

To address the practical limitations of EEN—such as poor palatability, social isolation, and the need for tube feeding—partial enteral nutrition (PEN) has been proposed as an alternative. This regimen combines a proportion of daily caloric intake from formula with regular food to improve acceptability and quality of life.

When used with an unrestricted diet, PEN alone has proven ineffective for inducing remission in CD [108]. However, emerging evidence suggests that PEN may achieve meaningful results when combined with structured exclusion diets.

Given the potential role of specific foods in perpetuating intestinal inflammation, targeted dietary protocols were developed to eliminate detrimental components. Among these, the CDED—created by Professor Arie Levine and colleagues—has shown particular promise. The CDED emphasizes the removal of foods known to disrupt the intestinal barrier or microbiota, such as processed products, emulsifiers, animal fats, red and processed meats, carrageenan, gluten, and dairy, while allowing whole, minimally processed items like chicken, eggs, rice, potatoes, fruits, and vegetables.

Because the CDED uses a standardized process, its ingredients are chosen to be widely available and reasonably priced. However, at first, local or traditional meals were not taken into account. There are numerous challenges that must be considered for CDED in the clinical practice, such as a lack of multidisciplinary teams and language barriers, a lack of information in the native language, lack of knowledge regarding healthy food and food items considered crucial in some cultural and social events. Therefore, CDED should be adapted to each tradition and culture, but it is imperative to uphold the fundamentals to maintain the core of CDED, as some cultures’ traditional food may not align with CDED. Over-modifications could cause a diet to deviate from the initial design, which could result in different results and misunderstandings regarding the effectiveness of CDED [109]. Although changes might accommodate regional tastes, they could lessen the diet’s effectiveness and undercut its actual potential. Therefore, care should be taken when designating such deviations as CDED, since they might not be in line with its intended design and might affect the results of the research. Cost was highlighted by Damas et al. as a deterrent to dietary adoption [110]. In a randomized pilot trial conducted in Argentina on 21 asymptomatic children with CD and elevated FC, CDED was followed with good compliance, and no diet adaptation was necessary [111]. In a retrospective study conducted by Landorf et al. in Australia on 24 pediatric subjects with CD, no cultural adaptations were described to increase CDED adherence, but adjustments were made for vegan diets and cow’s milk allergy [112]. In the 20th century, inflammatory bowel disease (IBD) was thought to be a condition that only affected early industrialized areas of Europe, North America and Oceania. IBD prevalence continued to rise steadily in early industrialized nations, but its incidence rose in newly industrialized and growing regions of Africa, Asia, and Latin America at the start of the twenty-first century. Epidemiologic data started to show that newly industrialized areas were starting to experience a period of sharply rising incidence at the turn of the twenty-first century. The evolution of IBD across epidemiologic stages helps healthcare systems better predict the future global burden of IBD. This increase in reported incidence can be attributed to both a true increase in incidence driven by environmental determinants and improved case identification through advanced diagnostic capabilities [113]. In South Africa, 9.7% of the 3278 IBD cases of the IBD Africa Registry, which is primarily based in Cape Town, have been documented in children and adolescents who were ≤18 years old at diagnosis. Children with IBD present a significant diagnostic difficulty in Africa. A significant clinical challenge is distinguishing IBD from common intestinal diseases such as intestinal TB, parasites, environmental enteropathy, chronic diarrhea, iron deficiency anemia, and failure to thrive as a result of chronic enteric infections. Additionally, pediatric IBD can manifest in a number of unique ways, which makes it more challenging to diagnose in clinics without specialized pediatric gastroenterology services [114].

In comparative trials, CDED combined with PEN was better tolerated than EEN in children with mild-to-moderate Crohn’s disease and demonstrated equivalent efficacy in inducing remission by week six. Furthermore, sustained remission has been achieved with this hybrid approach, suggesting it may offer a more sustainable alternative to strict EEN. In CDED + PEN, restrictive dietary interventions—if not properly balanced—may lead to energy and micronutrient deficiencies, thus requiring close medical and nutritional supervision [115]. Moreover, the commercial formulas commonly used in this protocol can be difficult to obtain or prohibitively expensive, especially within low-resource healthcare systems [116].

A summary of available data is presented in Table 3. The studies analyzed differ in the study design (Levine et al.’s study [70] is prospective, while Boneh et al.’s [117] and Niseteo et al.’s [118] studies are retrospective). The populations analyzed are also heterogeneous: Boneh et al. include both adults and children with loss of response to biologic therapy, whereas Levine et al.’s study involves exclusively pediatric patients with mild to moderate luminal CD who have not previously received biologic therapy. There are also differences in the endpoints used: some consider only clinical remission, while others include biomarkers and the microbiota in the variables considered.

### 5.4. Other Dietary Patterns in Pediatric Inflammatory Disease Treatment

In addition to EEN or PEN, several alternative dietary strategies have been explored for managing IBD, including the Specific Carbohydrate Diet (SCD), low-FODMAP diet, and Mediterranean Diet (MD). However, current evidence remains limited, and no formal consensus supports their routine clinical use in children with IBD due to insufficient data on safety and efficacy.

The SCD, first introduced in the early 20th century, restricts complex carbohydrates and refined sugars that are thought to promote bacterial overgrowth and dysbiosis, potentially exacerbating intestinal inflammation. The first prospective pediatric trial evaluating SCD in CD reported encouraging outcomes: at 12 weeks, 6 of 10 participants (60%) achieved clinical remission, with sustained remission in 6 of 7 patients who continued the diet for 52 weeks [121]. Further support came from the PRODUCE trial, which observed improvements in clinical indices, fecal calprotectin, and biochemical markers in patients aged 7–18 years with active IBD, accompanied by favorable changes in the intestinal microbiome, metabolome, and proteome [122]. Conversely, Wahbeh et al. found no evidence of mucosal healing with a modified SCD in pediatric CD, underscoring the need for additional prospective studies to confirm its long-term safety and therapeutic value [123]. Practically speaking, there are many obstacles to overcome while implementing the SCD and conformity. The diet’s restrictive nature, complexity, and requirement for extensive food preparation can impact quality of life and contribute to nutritional inadequacies if not properly monitored [124]. A study evaluated SCD’s nutritional effectiveness, finding that children with IBD on SCD generally consume enough of the majority of nutrients, but they show significant calcium and vitamin D deficits [125]. Therefore, patients who are on SCD need dietary recommendations, frequent monitoring, and possible supplements to guarantee adequate nutrition. Therefore, SCD is not currently supported by research as a primary dietary strategy for the treatment of IBD in pediatric patients.

The low-FODMAP diet (low in Fermentable Oligo-, Di-, Monosaccharides and Polyols) aims to reduce poorly absorbed carbohydrates that undergo bacterial fermentation, producing gas and short-chain fatty acids that may worsen gastrointestinal symptoms such as bloating, cramping, and diarrhea [126]. Evidence in pediatric IBD is scarce. A small case series of nine children with quiescent disease reported symptom improvement after a 4-week low-FODMAP intervention, with recurrence upon fructan challenge. Adult studies have not demonstrated efficacy for induction or maintenance of remission in CD or UC but suggest symptomatic benefit for functional symptoms in IBD patients [127,128].

The MD—rich in fruits, vegetables, legumes, nuts, whole grains, and olive oil, with moderate fish consumption and limited intake of red meat and dairy—has also been proposed for children with IBD. Strisciuglio et al. conducted a cross-sectional study in 125 pediatric patients (53 CD, 72 UC) and found that greater adherence to the MD correlated with significantly reduced fecal calprotectin levels [129]. Similarly, a 2022 randomized trial in 100 children with mild-to-moderate active IBD demonstrated that, after 12 weeks, those following the MD exhibited reductions in Pediatric Crohn’s Disease Activity Index (PCDAI), Pediatric Ulcerative Colitis Activity Index (PUCAI), C-reactive protein, calprotectin, tumor necrosis factor-α, and proinflammatory cytokines including interleukins 17, 12, and 13. Clinically speaking, the MD has various benefits when it comes to managing IBD. MD offers a balanced nutritional profile, higher than more restrictive diets, cultural acceptability, and culinary variety, which may improve long-term adherence. Additionally, its proven metabolic and cardiovascular advantages mitigate the elevated risk of comorbidities seen in people with IBD [130,131]. Because of these factors, the American Gastroenterological Association (AGA) suggests MD as an appropriate long-term dietary strategy for adult subjects with IBD, especially when they are in remission stages [132]. A study conducted by Lewis et al. compared SCD and MD’s effects in CD treatment in a cohort of 194 adult subjects with mild or moderate symptoms. In terms of symptomatic remission, fecal calprotectin level modifications, and CRP reduction, SCD was not better than MD. In light of these findings, the MD’s easier compliance, and additional health advantages, the MD might be chosen over the SCD for the majority of CD patients have mild to moderate symptoms [133]. Although these findings are promising, overall evidence remains preliminary, and further well-powered trials are needed to confirm the MD’s therapeutic potential in pediatric IBD.

Finally, several ongoing studies are investigating other dietary modifications—such as the exclusion of ultra-processed foods, red or processed meat, and cow’s milk proteins—but most are small and heterogeneous, precluding firm conclusions at this time [134,135].

Practical points to apply when dealing with pediatric patients with IBD, according to ESPGHAN guidelines [72], are summarized in Table 4.

## 6. Nutritional Risks for Subjects with pIBD

Malnutrition encompasses any state of nutritional imbalance—whether due to insufficient or excessive nutrient intake—resulting in alterations of body composition, particularly a loss of lean mass, and subsequent impairment of physical, cognitive, and metabolic functions. While commonly associated with undernutrition, the term also includes conditions of overnutrition, such as obesity accompanied by micronutrient deficiency [136].

The prevalence of malnutrition among individuals with IBD may reach up to 85% [67]. Although it occurs in both CD and UC, it is more frequent in CD because the disease can affect any portion of the gastrointestinal tract, leading to impaired nutrient absorption. In contrast, UC—limited to the colon—has less direct impact on absorptive capacity [137].

The development of malnutrition in IBD is multifactorial. Inflammatory activity disrupts intestinal barrier function, diminishing absorption of macronutrients, vitamins, and minerals, while proinflammatory cytokines such as TNF-α and IL-6 elevate resting energy expenditure and drive a catabolic state that promotes muscle wasting and weight loss. Additionally, nutrient deficiencies can alter the gut microbiota, perpetuating dysbiosis, which in turn exacerbates intestinal inflammation and sustains a self-reinforcing cycle of nutritional and inflammatory deterioration [138,139].

In pediatric patients, the long-term effects of malnutrition are particularly significant, encompassing linear growth failure, altered body composition, delayed puberty, bone demineralization, anemia, obesity, and psychosocial disturbances, including disordered eating behaviors [72].

### 6.1. Linear Growth Impairment

Growth retardation is a well-recognized complication in children with IBD—most notably in CD—and may precede diagnosis or persist despite apparent disease control [10]. Studies estimate that 11–35% of affected children fail to reach their genetic height potential, with an average shortfall of around 7 cm [140,141]. In UC, growth impairment is less common (5–12%) and often improves with effective therapy [142].

Contributing factors include:

Chronic inflammation: Proinflammatory cytokines (TNF-α, IL-6) interfere with the GH/IGF-1 axis, suppressing linear growth [143].

- Nutritional deficiencies: Reduced intake due to pain, anorexia, or dietary restriction, combined with malabsorption, leads to deficits in essential growth nutrients [143,144].
- Increased energy expenditure: Inflammation-driven hypermetabolism further aggravates undernutrition [144].
- Delayed puberty: Malnutrition and inflammation postpone the pubertal growth spurt [143].
- Genetic predisposition: Parental height and IBD-associated polymorphisms influence growth potential [142].
- Corticosteroid therapy: Prolonged use directly impairs the growth plate and bone metabolism [143].

Lee et al. demonstrated that final adult height in PIBD is primarily predicted by the genetic target height and the lowest recorded height-for-age Z-score during illness. With early diagnosis, optimized therapy, and careful monitoring, most children can attain near-normal adult stature, whereas those with prolonged growth suppression remain at greater risk for permanent short stature [141].

### 6.2. Altered Body Composition

Children with IBD frequently present with reduced lean body mass even when total weight or BMI appears normal. A 2015 systematic review confirmed this consistent pattern, often accompanied by increased fat mass—likely reflecting the combined effects of inflammation, nutrient deficiency, and corticosteroid therapy [145]. These findings underscore the limitations of relying solely on BMI and highlight the need for comprehensive body composition assessments when evaluating nutritional status [146].

### 6.3. Weight Excess

Contrary to traditional assumptions, overweight and obesity are increasingly recognized among pediatric IBD patients [147,148]. These data challenge the historical perception of underweight as the predominant nutritional issue at diagnosis. Obesity and IBD share a bidirectional relationship: adiposity contributes to systemic inflammation, microbiota alterations, and immune dysregulation, all of which may exacerbate disease activity. Moreover, excess weight can impair therapeutic response—particularly to biologics—and is associated with greater complication rates [149].

### 6.4. Delayed Puberty

Delayed pubertal development is a common extraintestinal complication, especially in CD [150]. Although malnutrition is a major contributing factor, inflammatory cytokines may also disrupt the hypothalamic–pituitary–gonadal axis. Notably, serum androgen levels are often decreased in affected patients, reflecting both nutritional and inflammatory influences on pubertal progression [151].

### 6.5. Impaired Bone Mineralization

Low bone mineral density (BMD) is frequently observed in pediatric CD and, to a lesser extent, UC, predisposing patients to fractures and early-onset osteoporosis [151]. Contributing factors include chronic inflammation, vitamin D deficiency, and long-term corticosteroid exposure [151,152,153]. In a 2014 randomized trial, weekly risedronate combined with calcium and vitamin D supplementation significantly improved lumbar spine BMD in children with CD-related osteopenia [154].

### 6.6. Anemia

Anemia is among the most prevalent complications in pediatric IBD [67]. It commonly manifests as iron-deficiency anemia, anemia of chronic disease, or mixed forms [155]. Routine hematologic screening is recommended every three months during active disease and every 6–12 months during remission [155]. Iron supplementation should be initiated when deficiency is confirmed, as correction of anemia enhances quality of life independently of inflammatory control [155].

### 6.7. Psychosocial Aspects and Eating Disorders

Children and adolescents with IBD are at heightened risk for psychological distress, including anxiety, depression, and maladaptive eating behaviors [156,157]. Qualitative research by Czuber-Dochan et al. revealed that disease-related changes in appetite, pain, and dietary restrictions often foster food-related anxiety and social withdrawal [157]. Although perceived disease stigma is linked to more depressed symptoms in young people with IBD, it is still unknown how stigma affects emotional adjustment. When faced with uncertainty about their illness, young people with IBD who are more present-focused and more able to accept the less controllable parts of their condition (i.e., are aware) may develop more adaptive techniques, leading to more positive emotional adjustment. The detrimental effects of stigma and illness uncertainty on adjustment in this population may be lessened by clinical therapies that prioritize mindfulness training in addition to recognition and acceptance of IBD illness characteristics [158]. The transition from childhood to adult age is another delicate moment as well. Patients with pIBD, particularly those between the ages of 12 and 17, are ill-prepared for the shift to adult treatment. Although there is a decent amount of generic IBD management awareness, specific educational initiatives are required. Reliable information sources and early, individualized counseling services could improve patient outcomes, long-term disease management, and transition experiences [159]. A recent study analyzed the disease’s burden on families of pediatric patients through a detailed questionnaire. Parents of children with IBD are also affected by the illness burden. Parents of IBD patients are more concerned about possible adverse effects from IBD drugs and are reluctant to have their children take part in drug-related clinical trials [160]. Many patients develop restrictive or avoidant eating habits driven by fear of symptom exacerbation, which can perpetuate nutritional deficits and impair psychosocial well-being. Integrating nutritional counseling with psychological support is therefore essential for comprehensive pediatric IBD management. In this context, multidisciplinary support, including psychologists and social workers, would be the ideal setting in order to improve patients’ adherence to prescribed therapy and to tailor support to them and their families.

## 7. Conclusions

PIBD represents a multifaceted clinical challenge, encompassing both diagnostic complexity and long-term management difficulties. Disease onset during childhood or adolescence is frequently characterized by more extensive and aggressive phenotypes compared with adult-onset forms, with significant consequences for growth, pubertal development, bone health, and overall quality of life. A multidisciplinary, patient-centered approach—involving pediatric gastroenterologists, dietitians, psychologists, and other allied health professionals—is therefore essential to ensure comprehensive and individualized care.

In IBD, a peripheral pro-glycolytic inflammatory environment is triggered by increased intestinal permeability and microbial translocation. Inflammation and immune cellular metabolic reprogramming are characteristics of the intestinal tight junction. The pathogenesis of obesity, cancer, and aging disorders may also be significantly influenced by a compromised gut barrier and immune cell metabolic remodeling. This may be the link that connects different diseases that can cause long-term catabolic consequences, and this hypothesis will surely be tested in further studies.

Although the therapeutic landscape has advanced substantially with the introduction of biologic and targeted agents, nutritional therapy remains a cornerstone of PIBD management. EEN and selective exclusion diets offer effective, steroid-sparing alternatives for inducing remission and promoting mucosal healing, while supporting growth and nutritional recovery. Personalized nutritional interventions, combined with optimized pharmacological treatment, represent the current gold standard for improving outcomes and minimizing treatment-related adverse effects.

EEN remains the preferred dietary therapy for pediatric CD, supported by robust evidence of efficacy and safety. However, its implementation is often limited by challenges such as dietary monotony, high formula cost, and the social burden of nasogastric feeding or prolonged food restriction. Regular involvement of a specialized dietitian is crucial to ensure adequate caloric, macronutrient, and micronutrient intake and to support adherence. Despite its clinical success, the mechanisms underlying nutritional therapy—including its effects on the microbiome, immune modulation, and intestinal barrier integrity—are not yet fully elucidated. Future research should aim to clarify these mechanisms, establish standardized dietary protocols, and define predictors of response to optimize the use of diet-based strategies. The growing body of evidence highlights the transformative potential of nutritional therapy in PIBD, particularly CD. Continued high-quality studies are needed to confirm its efficacy, enhance adherence strategies, and integrate dietary management as a fundamental component of precision medicine for young patients living with IBD.

## Figures and Tables

**Table 1:** Pharmacologic Therapies in Pediatric Inflammatory Bowel Disease.

Drug	Indication	Pediatric Dosage	Administration Interval	Main Side Effects
Prednisone	Induction (CD & UC)	1–2 mg/kg/day (max 40–60 mg/day), taper over 6–8 weeks	Daily oral	Growth suppression, weight gain, mood changes, osteoporosis
Budesonide	Induction (mild CD)	9 mg/day orally (or 6 mg/m^2^/day)	Daily oral	Less systemic effect, adrenal suppression, GI symptoms
Azathioprine (AZA)	Maintenance	1.5–2.5 mg/kg/day	Daily oral	Leukopenia, hepatotoxicity, pancreatitis, lymphoma risk
6-Mercaptopurine	Maintenance	1–1.5 mg/kg/day	Daily oral	Like AZA
Methotrexate (MTX)	Maintenance (esp. CD)	15 mg/m^2^/week (max 25 mg), subcutaneous or oral	Weekly	Nausea, liver toxicity, bone marrow suppression
Infliximab (IFX)	Induction and maintenance (CD and UC)	5 mg/kg induction at 0, 2, 6 weeks 5 m/kg 8 weekly for maintenance	Weeks 0, 2, 6, then every 8 weeks	Infusion reactions, infections, antibodies, psoriasis
Adalimumab (ADA)	Induction and maintenance (CD)	>40 kg = 80 mg + 40 mg for induction at 0, 2 weeks and 40 mg 2 weekly for maintenance < 40 kg, induction dosing is 40 mg + 20 mg at 0, 2 weeks and 20 mg 2 weekly for maintenance	Subcutaneous injection	Injection site reactions, infections, antibody formation
Vedolizumab	Refractory CD & UC	6 mg/kg, max dose of 300 mg Induction given at 0, 2 and 6 weeks Maintenance every 8 weeks Subcutaneous dosing may be considered (2 mg/kg subcutaneous 2 weekly after intravenous)	Weeks 0, 2, 6, then every 8 weeks	Mild: headache, fatigue, URTI; rare PML
Ustekinumab	Moderate-severe CD	<40 kg = 6 mg/kg intravenous infusion for induction followed by 45 mg subcutaneous every 8 weeks for maintenance in children > 40 kg = 390 mg intravenous infusion for induction followed by 90 mg subcutaneous every 8 weeks	IV once, then SC maintenance	URTI, injection site reaction, headache

CD, Chron’s disease; IV, intravenous; PML, progressive multifocal leukoencephalopathy; SC, subcutaneous; UC, ulcerative colitis; URTI, upper respiratory tract infection. Adapted from references [67,68,69,70].

**Table 2:** Exclusive enteral nutrition in pediatric subjects with inflammatory bowel disease.

Author	Year of Publication	Patients Included	Duration of Treatment	Type of Feed	Remission Rate	Comments
Levine et al. [70]	2014	201	6–8 weeks	Polymeric	86.6%	No difference between EEN and CS
Luo et al. [97]	2015	28	8 weeks	Polymeric	90%	The remission rate in EEN group was significantly higher than that in CS group (90.0% vs. 50.0%, respectively, *p* < 0.05).
Soo et al. [96]	2013	105	6–8 weeks	Polymeric	88.9%	Remission rate was similar for EEN group and CS group (91.3%)
MacLellan et al. [94]	2017	111	6–16 weeks	Unknown	86.6%	Induction with EEN was significantly associated with reduced risk of exposure to CS over 2 years
Pigneur et al. [95]	2019	19	8 weeks	Polymeric	89%	Clinical remission and biological markers are similar in both groups (EEN and CS). EEN induces better mucosal healing. There is also a difference in the microbiome compositing after the two types of treatments

CS, corticosteroids; EEN, exclusive enteral nutrition.

**Table 3:** Partial Enteral Nutrition in IBD. CD, Crohn’s disease; CDED, Crohn’s disease exclusion diet; EEN, exclusive enteral nutrition; PEN, partial enteral nutrition.

Author	Year of Publication	Patients Included	Type of Feed	Comments
Levine et al. [70]	2019	4–18 years; mild to moderate luminal CD (PCDAI > 10 and <40) and evidence for active inflammation	Group 1: CDED plus 50% of calories from formula for 6 weeks followed by CDED with 25% PEN from weeks 7 to 12; Group 2: EEN for 6 weeks followed by a free diet with 25% PEN from weeks 7 to 12	Clinical remission with CDED + PEN was 75%; with EEN was 59% at week 6; CDED + PEN was superior to EEN group at 12 weeks in all clinical parameters
Niseteo et al. [118]	2021	Children who were newly diagnosed with CD	Group 1: EEN for 6–8 weeks. Group 2: CDED + PEN for 6–8 weeks (80% of patients initially received EEN for 1–2 weeks).	CDED + PEN (with prior 1–2 weeks of EEN) has comparable efficacy to EEN therapy in inducing remission (75% remission rate). CDED + PEN leads to better weight gain.
Matuszczyk et al. [119]	2022	4–17 years; mild-moderate CD children with elevated FC	CDED plus 50% PEN for 6 weeks followed by CDED with 25% PEN for another 6 weeks	12 weeks of CDED + PEN has a beneficial effect on the fecal calprotectin
María Clara Jijón Andrade et al. [120]	2023	Children aged 10.7–15 years with new onset mild-moderate and loss of response to biologics	CDED + PEN for 24 weeks	CDED + PEN among 15 patients with CD resulted in remission in all patients at weeks 6 and 12. In naive patients, 87% maintained remission at week 24 vs. 67% of patients who lost response to biologics. In naïve patients the FCP decreased at week 6, week 12, and week 24, whereas in patients who lost response to biologics, the reduction in FCP was not significant.
Sigall-Boneh et al. [117]	2017	CD patients (<20 years) with loss of response to biologics	CDED + 50% PEN for 6 weeks followed by CDED with 25% PEN for another 6 weeks, or CDED alone. Severe patients started with EEN for 2 weeks and continued with CDED plus PEN	Clinical response and remission were achieved in 90% and 62%, respectively, and led to improvement in inflammatory markers at week 6

**Table 4:** Nutritional treatment of inflammatory bowel disease (IBD) in clinical practice, adapted from [72].

Oral Nutrition	
Issue	Clinical Practice
Macronutrients and energy needs	In the case of insufficient intake, dietary consultation is recommended. The general intake may be increased on an individual basis by increasing food intake and food fortification. If those are not enough, a supplemental formula or supplements may be recommended. Consider nasogastric feed if nutritional needs cannot be met orally.
Protein requirement	During active disease with poor nutritional state, it may be recommended to increase protein intake by at least 25% initially, or until linear growth has improved.
Minerals and vitamins	It seems prudent to assess zinc status during prolonged diarrheal episodes (>4 weeks). When zinc deficiency is encountered, a short course (2–4 weeks) of oral zinc is usually sufficient to restore adequate serum levels. Oral iron replacement is associated with high levels of intolerance and consequent lack of adherence. IV ferric carboxymaltose supplementation is better tolerated and superior to oral treatment, particularly in active IBD with low hemoglobin < 10 g/dL. A specific dose for calcium supplementation is not recommended. A standard weight-based dose for replenishment of vitamin D in children with IBD has not yet been established. Evidence implies that high doses (i.e., ≥2000 UI daily or 50,000 IU weekly) and long-term treatment may, however, be necessary to maintain sufficiency. Folate supplementation of 1 mg/day is usually sufficient to replenish deficient folate stores within 2 to 3 weeks; however, the folic acid requirement of children with CD has not been determined. Patients with extensive distal ileal resection or with clinical deficiency of vitamin B12 should receive 1000 mg of injected B12 every other day for 1 week and then weekly until clinically improved, followed by periodic injections according to methylmalonic levels.
**Nutritional Therapy**	
Parenteral Nutrition (PN)	PN should only be used when oral nutrition support or enteral support is insufficient to meet the patients’ requirements or when enteral feeding is contraindicated. PN is more expensive and raises safety concerns in comparison to EEN.
Exclusive Enteral Nutrition (EEN)	There is no evidence that the dietary source of proteins affects the efficacy of EEN. Initially the formula should be administered orally. A nasogastric tube may be used when there is failure to achieve adequate oral intake. Recommended for at least 8 weeks. There is still insufficient evidence to recommend a standard food reintroduction scheme. In the absence of evidence we suggest a gradual reintroduction of the foods, with a concomitant reduction in the formula over a 2- to 3-week period. There is insufficient evidence to recommend EEN for isolated oral or perianal disease and for extraintestinal manifestations.
Partial Enteral Nutrition (PEN)	PEN alone is not efficacious to induce remission in the majority of patients. Supplementation with a standard polymeric formula, in addition to conventional induction treatment, may be considered.
Probiotics and Prebiotics	Probiotics should be used with caution in patients with central venous catheter or in immunocompromised patients. Results from clinical trials are strain-specific and should not be extrapolated to other bacterial strains.

## Abbreviations

AGA	American Gastroenterological Association
ASCAs	Anti-Saccharomyces cerevisiae antibodies
BMI	Body mass index
CD	Chron’s disease
CDED	Crohn’s disease exclusion diet
CS	Corticosteroids
CRP	C-reactive protein
DALYs	Disability-adjusted life years
EEN	Exclusive enteral nutrition
ESPEN	European Society for Clinical Nutrition and Metabolism
ESPGHAN	European Society of Pediatric Gastroenterology, Hepatology, and Nutrition
ESR	Erythrocyte sedimentation rate
FC	Fecal calprotectin
FMT	Fecal microbiota transplantation
FODMAP	Fermentable Oligo-, Di-, Monosaccharides, and Polyols
GI	Gastrointestinal
HBI	Harvey–Bradshaw index
IBD	Inflammatory bowel disease
IBD-U	Inflammatory bowel disease unclassified
ILC3s	Group 3 innate lymphoid cells
IL-22	Interleukin-22
MAMPs	Microbe-associated molecular patterns
MD	Mediterranean diet
MRE	Magnetic resonance enterography
NLRs	NOD-like receptors
pANCAs	Perinuclear anti-neutrophil cytoplasmic antibodies
PAMPs	Pathogen-associated molecular patterns
PEN	Partial enteral nutrition
PIBD	Pediatric-onset inflammatory bowel disease
PID	Primary immunodeficiencies
REE	Resting energy expenditure
SCD	Specific carbohydrate diet
SCFAs	Short-chain fatty acids
SDI	Socio-demographic index
Th17	T helper 17
TLRs	Toll-like receptors
TNFalpha	Tumor necrosis factor-alpha
UC	Ulcerative colitis
VEO-IBD	Very-early-onset IBD
WCE	Wireless capsule endoscopy

## References

[1] Carroll M.W., Kuenzig M.E., Mack D.R., Otley A.R., Griffiths A.M., Kaplan G.G., Bernstein C.N., Bitton A., Murthy S.K., Nguyen G.C. (2019). The impact of inflammatory bowel disease in Canada 2018: Children and adolescents with IBD. J. Can. Assoc. Gastroenterol..

[2] Kuenzig M.E., Fung S.G., Marderfeld L., Mak J.W.Y., Kaplan G.G., Ng S.C., Wilson D.C., Cameron F., Henderson P., Kotze P.G. (2022). Twenty-first century trends in the global epidemiology of pediatric-onset inflammatory bowel disease: Systematic review. Gastroenterology.

[3] Long D., Wang C., Huang Y., Mao C., Xu Y., Zhu Y. (2024). Changing epidemiology of inflammatory bowel disease in children and adolescents. Int. J. Color. Dis..

[4] Wang Y., Pan C.-W., Huang Y., Zheng X., Li S., He M., Hashash J.G., A Farraye F., Ehrlich A.C. (2025). Global epidemiology and geographic variations of pediatric-onset inflammatory bowel disease: A comprehensive analysis of the Global Burden of Disease Study 1990 to 2019. Inflamm. Bowel Dis..

[5] Roberts S.E., Thorne K., Thapar N., Broekaert I., Benninga M.A., Dolinsek J., Mas E., Miele E., Orel R., Pienar C. (2020). A systematic review and meta-analysis of paediatric inflammatory bowel disease incidence and prevalence across Europe. J. Crohns Colitis.

[6] Alvisi P., Labriola F., Scarallo L., Gandullia P., Knafelz D., Bramuzzo M., Zuin G., Pastore M.R., Illiceto M.T., Miele E. (2022). Epidemiological trends of pediatric IBD in Italy: A 10-year analysis of the Italian society of pediatric gastroenterology, hepatology and nutrition registry. Dig. Liver Dis..

[7] Khor B., Gardet A., Xavier R.J. (2011). Genetics and pathogenesis of inflammatory bowel disease. Nature.

[8] Scheffers L.E., Vos I.K., Utens E.M.W.J., Dieleman G.C., Walet S., Escher J.C., van den Berg L.E.M., Rotterdam Exercise Team (2023). Physical Training and Healthy Diet Improved Bowel Symptoms, Quality of Life, and Fatigue in Children with Inflammatory Bowel Disease. J. Pediatr. Gastroenterol. Nutr..

[9] Ng S.C., Shi H.Y., Hamidi N., Underwood F.E., Tang W., Benchimol E.I., Panaccione R., Ghosh S., Wu J.C.Y., Chan F.K.L. (2017). Worldwide incidence and prevalence of inflammatory bowel disease in the 21st century: A systematic review of population-based studies. Lancet.

[10] Torres J., Mehandru S., Colombel J.-F., Peyrin-Biroulet L. (2017). Crohn’s disease. Lancet.

[11] Ungaro R., Mehandru S., Allen P.B., Peyrin-Biroulet L., Colombel J.-F. (2017). Ulcerative colitis. Lancet.

[12] Bouhuys M., Lexmond W.S., van Rheenen P.F. (2023). Pediatric inflammatory bowel disease. Pediatrics.

[13] Uhlig H.H., Charbit-Henrion F., Kotlarz D., Shouval D.S., Schwerd T., Strisciuglio C., de Ridder L., van Limbergen J., Macchi M., Snapper S.B. (2021). Clinical genomics for the diagnosis of monogenic forms of inflammatory bowel disease. J. Pediatr. Gastroenterol. Nutr..

[14] Molodecky N.A., Kaplan G.G. (2010). Environmental risk factors for inflammatory bowel disease. Gastroenterol. Hepatol..

[15] Jostins L., Ripke S., Weersma R.K., Duerr R.H., McGovern D.P., Hui K.Y., Lee J.C., Schumm L.P., Sharma Y., Anderson C.A. (2012). Host-microbe interactions have shaped the genetic architecture of inflammatory bowel disease. Nature.

[16] Ni J., Wu G.D., Albenberg L., Tomov V.T. (2017). Gut microbiota and IBD: Causation or correlation?. Nat. Rev. Gastroenterol. Hepatol..

[17] Levine A., Koletzko S., Turner D., Escher J.C., Cucchiara S., de Ridder L., Kolho K., Veres G., Russell R.K., Paerregaard A. (2014). ESPGHAN revised porto criteria for the diagnosis of inflammatory bowel disease in children and adolescents. J. Pediatr. Gastroenterol. Nutr..

[18] Torres J., Bonovas S., Doherty G., Kucharzik T., Gisbert J.P., Raine T., Adamina M., Armuzzi A., Bachmann O., Bager P. (2020). ECCO guidelines on therapeutics in crohn’s disease: Medical treatment. J. Crohns Colitis.

[19] Uhlig H.H., Schwerd T., Koletzko S., Shah N., Kammermeier J., Elkadri A., Ouahed J., Wilson D.C., Travis S.P., Turner D. (2014). The diagnostic approach to monogenic very early onset inflammatory bowel disease. Gastroenterology.

[20] Levine A., Griffiths A., Markowitz J. (2011). Pediatric modification of the Montreal classification for IBD: The Paris classification. Inflamm. Bowel Dis..

[21] Bousvaros A., Antonioli D.A., Colletti R.B., Dubinsky M.C., Glickman J.N., Gold B.D., Griffiths A.M., Jevon G.P., North American Society for Pediatric Gastroenterology, Hepatology, and Nutrition, Colitis Foundation of America (2007). Differentiating ulcerative colitis from Crohn disease in children and young adults: Report of a working group of the North American Society for Pediatric Gastroenterology, Hepatology, and Nutrition and the Crohn’s and Colitis Foundation of America. J. Pediatr. Gastroenterol. Nutr..

[22] de Bie C.I., Buderus S., Sandhu B.K., de Ridder L., Paerregaard A., Veres G., Dias J.A., Escher J.C., EUROKIDS Porto IBD Working Group of ESPGHAN (2012). Diagnostic workup of paediatric patients with inflammatory bowel disease in Europe: Results of a 5-year audit of the EUROKIDS registry. J. Pediatr. Gastroenterol. Nutr..

[23] Gupta N., Bostrom A.G., Kirschner B.S., Ferry G.D., Gold B.D., Cohen S.A., Winter H.S., Baldassano R.N., Abramson O., Smith T. (2010). Incidence of stricturing and penetrating complications of Crohn’s disease diagnosed in pediatric patients. Inflamm. Bowel Dis..

[24] Odze R.D., Bines J., Leichtner A.M., Goldman H., Antonioli D.A. (1993). Allergic proctocolitis in infants: A prospective clinicopathologic biopsy study. Hum. Pathol..

[25] Van Limbergen J., Russell R.K., Drummond H.E. (2008). Definition of phenotypic characteristics of childhood-onset IBD. Gastroenterology.

[26] IBD Working Group of the European Society for Paediatric Gastroenterology, Hepatology and Nutrition (2005). Inflammatory bowel disease in children and adolescents: Recommendations for diagnosis-the Porto criteria. J. Pediatr. Gastroenterol. Nutr..

[27] Ruemmele F.M., Veres G., Kolho K.L., Griffiths A., Levine A., Escher J.C., Amil Dias J., Barabino A., Braegger C.P., Bronsky J. (2014). Consensus guidelines of ECCO/ESPGHAN on the medical management of pediatric Crohn’s disease. J. Crohns Colitis.

[28] Martín-de-Carpi J., Rodríguez A., Ramos E., Jiménez S., Martínez-Gómez M.J., Medina E., SPIRIT-IBD Working Group of Sociedad Española de Gastroenterología, Hepatología y Nutricion Pediátrica (2013). Increasing incidence of pediatric inflammatory bowel disease in Spain (1996-2009): The SPIRIT Registry. Inflamm. Bowel Dis..

[29] Henderson P., Anderson N.H., Wilson D.C. (2014). The diagnostic accuracy of fecal calprotectin during the investigation of suspected pediatric inflammatory bowel disease: A systematic review and meta-analysis. Am. J. Gastroenterol..

[30] Fagerberg U.L., Lööf L., Lindholm J., Hansson L.O., Finkel Y. (2007). Fecal calprotectin: A quantitative marker of colonic inflammation in children with IBD. J. Pediatr. Gastroenterol. Nutr..

[31] Van Rheenen P.F., Van De Vijver E., Fidler V. (2010). Faecal calprotectin for screening of patients with suspected IBD: Diagnostic meta-analysis. BMJ.

[32] Hyams J., Markowitz J., Otley A., Rosh J., Mack D., Bousvaros A., Kugathasan S., Pfefferkorn M., Tolia V., Evans J. (2005). Evaluation of the pediatric crohn disease activity index: A prospective multicenter experience. J. Pediatr. Gastroenterol. Nutr..

[33] Kellar A., Chavannes M., Huynh H.Q., Aronskyy I., Lei B., deBruyn J.C., Kim J., Dolinger M.T. (2025). Defining normal bowel wall thickness in children with inflammatory bowel disease in deep remission: A multicenter study on behalf of the pediatric committee of the International Bowel Ultrasound Group (IBUS). J. Pediatr. Gastroenterol. Nutr..

[34] Harvey R.F., Bradshaw J.M. (1980). A simple index of Crohn’s-disease activity. Lancet.

[35] de Souza H.S.P., Fiocchi C. (2016). Immunopathogenesis of IBD: Current state of the art. Nat. Rev. Gastroenterol. Hepatol..

[36] Piovani D., Danese S., Peyrin-Biroulet L., Nikolopoulos G.K., Lytras T., Bonovas S. (2019). Environmental Risk Factors for Inflammatory Bowel Diseases: An Umbrella Review of Meta-analyses. Gastroenterology.

[37] Ananthakrishnan A.N. (2015). Environmental risk factors for inflammatory bowel diseases: A review. Dig. Dis. Sci..

[38] Hou J.K., Abraham B., El-Serag H. (2011). Dietary intake and risk of developing inflammatory bowel disease: A systematic review of the literature. Am. J. Gastroenterol..

[39] Sevelsted A., Stokholm J., Bønnelykke K., Bisgaard H. (2015). Cesarean section and chronic immune disorders. Pediatrics.

[40] Hviid A., Svanström H., Frisch M. (2011). Antibiotic use and inflammatory bowel diseases in childhood. Gut.

[41] Barclay A.R., Russell R.K., Wilson M.L., Gilmour W.H., Satsangi J. (2009). Systematic review: The role of breastfeeding in the development of paediatric inflammatory bowel disease. J. Pediatr. Gastroenterol. Nutr..

[42] Capra M.E., Aliverti V., Bellani A.M., Berzieri M., Montani A.G., Pisseri G., Sguerso T., Esposito S., Biasucci G. (2025). Breastfeeding and Non-Communicable Diseases: A Narrative Review. Nutrients.

[43] Azad M.B., Konya T., Maughan H., Guttman D.S., Field C.J., Sears M.R., Becker A.B., Scott J.A., Kozyrskyj A.L., CHILD Study Investigators (2013). Infant gut microbiota and the hygiene hypothesis of allergic disease: Impact of household pets and siblings on microbiota composition and diversity. Allergy Asthma Clin. Immunol..

[44] Capra M.E., Monopoli D., Decarolis N.M., Giudice A., Stanyevic B., Esposito S., Biasucci G. (2023). Dietary Models and Cardiovascular Risk Prevention in Pediatric Patients. Nutrients.

[45] Capra M.E., Biasucci G., Travaglia E., Sodero R., Banderali G., Pederiva C. (2025). Fiber in the Treatment of Dyslipidemia in Pediatric Patients. Children.

[46] Fitzgerald R.S., Sanderson I.R., Claesson M.J. (2021). Paediatric inflammatory bowel disease and its relationship with the microbiome. Microb. Ecol..

[47] Caruso R., Lo B.C., Núñez G. (2020). Host-microbiota interactions in inflammatory bowel disease. Nat. Rev. Immunol..

[48] Wang S.-L., Wang Z.-R., Yang C.-Q. (2012). Meta-analysis of broad-spectrum antibiotic therapy in patients with active inflammatory bowel disease. Exp. Ther. Med..

[49] Cococcioni L., Panelli S., Varotto-Boccazzi I., Carlo D.D., Pistone D., Leccese G., Zuccotti G.V., Comandatore F. (2021). IBDs and the pediatric age: Their peculiarities and the involvement of the microbiota. Dig. Liver Dis..

[50] Imhann F., Vila V., Bonder A. (2018). Interplay of host genetics and gut microbiota underlying the onset and clinical presentation of inflammatory bowel disease. Gut.

[51] Honda K., Littman D.R. (2016). The microbiota in adaptive immune homeostasis and disease. Nature.

[52] Kostic A.D., Xavier R.J., Gevers D. (2014). The microbiome in inflammatory bowel disease: Current status and the future ahead. Gastroenterology.

[53] Sartor R.B. (2008). Microbial influences in inflammatory bowel diseases. Gastroenterology.

[54] Britton G.J., Contijoch E.J., Mogno I., Vennaro O.H., Llewellyn S.R., Ng R., Li Z., Mortha A., Merad M., Das A. (2019). Microbiotas from Humans with Inflammatory Bowel Disease Alter the Balance of Gut Th17 and RORγt + Regulatory T Cells andExacerbate Colitis in Mice. Immunity.

[55] Abdel-Rahman L.I.H., Morgan X.C. (2023). Searching for a Consensus Among Inflammatory Bowel Disease Studies: A Systematic Meta-Analysis. Inflamm. Bowel Dis..

[56] Sugihara K., Kamada N. (2024). Metabolic Network of the Gut Microbiota in Inflammatory Bowel Disease. Inflamm. Regen..

[57] Leibovitzh H., Lee S.-H., Xue M., Raygoza Garay J.A., Hernandez-Rocha C., Madsen K.L., Meddings J.B., Guttman D.S., EspinGarcia O., Smith M.I. (2022). Altered Gut Microbiome Composition and Function Are Associated with Gut Barrier Dysfunction in Healthy Relatives of Patients with Crohn’s Disease. Gastroenterology.

[58] Round J.L., Mazmanian S.K. (2009). The gut microbiota shapes intestinal immune responses during health and disease. Nat. Rev. Immunol..

[59] Oliva S., Thomson M., de Ridder L., Martín-de-Carpi J., Van Biervliet S., Braegger C., Dias J.A., Kolacek S., Miele E., Buderus S. (2018). Endoscopy in pediatric inflammatory bowel disease: A position paper on behalf of the Porto IBD group of the European society for pediatric gastroenterology, hepatology and nutrition. J. Pediatr. Gastroenterol. Nutr..

[60] Guandalini S. (2024). Probiotics in the treatment of inflammatory bowel diseases. Adv. Exp. Med. Biol..

[61] Uhlig H.H., Schwerd T. (2016). From Genes to Mechanisms: The Expanding Spectrum of Monogenic Disorders Associated with Inflammatory Bowel Disease. Inflamm. Bowel Dis..

[62] de Lange K.M., Moutsianas L., Lee J.C., Lamb C.A., Luo Y., Kennedy N.A., Jostins L., Rice D.L., Gutierrez-Achury J., Ji S.-G. (2017). Genome-wide association study implicates immune activation of multiple integrin genes in inflammatory bowel disease. Nat. Genet..

[63] Ouahed J., Spencer E., Kotlarz D., Shouval D.S., Kowalik M., Peng K., Field M., Grushkin-Lerner L., Pai S.Y., Bousvaros A. (2020). Very Early Onset Inflammatory Bowel Disease: A Clinical Approach with a Focus on the Role of Genetics and Underlying Immune Deficiencies. Inflamm. Bowel Dis..

[64] Zeissig Y., Petersen B.-S., Milutinovic S., Bosse E., Mayr G., Peuker K., Hartwig J., Keller A., Kohl M., Laass M.W. (2015). XIAP variants in male Crohn’s disease. Gut.

[65] Alarfaj S.J., Mostafa S.A., Negm W.A., El-Masry T.A., Kamal M., Elsaeed M., El Nakib A.M. (2023). Mucosal Genes Expression in Inflammatory Bowel Disease Patients: New Insights. Pharmaceuticals.

[66] Kelsen J.R., Baldassano R.N., Artis D., Sonnenberg G.F. (2015). Maintaining intestinal health: The genetics and immunology of very early onset inflammatory bowel disease. Cell. Mol. Gastroenterol. Hepatol..

[67] Vuijk S.A., Camman A.E., de Ridder L. (2024). Considerations in paediatric and adolescent inflammatory bowel disease. J. Crohns Colitis.

[68] Hyams J.S., Griffiths A., Markowitz J., Baldassano R.N., Faubion W.A., Colletti R.B., Dubinsky M., Kierkus J., Rosh J., Wang Y. (2012). Safety and efficacy of adalimumab for moderate to severe Crohn’s disease in children. Gastroenterology.

[69] Ashton J.J., Beattie R.M. (2024). Inflammatory bowel disease: Recent developments. Arch. Dis. Child..

[70] Levine A., Wine E., Assa A., Sigall Boneh R., Shaoul R., Kori M., Cohen S., Peleg S., Shamaly H., On A. (2019). Crohn’s disease exclusion diet plus partial enteral nutrition induces sustained remission in a randomized controlled trial. Gastroenterology.

[71] Roncoroni L., Gori R., Elli L., Tontini G.E., Doneda L., Norsa L., Cuomo M., Lombardo V., Scricciolo A., Caprioli F. (2022). Nutrition in patients with inflammatory bowel diseases: A narrative review. Nutrients.

[72] Crooks B., McLaughlin J., Matsuoka K., Kobayashi T., Yamazaki H., Limdi J.K. (2021). The dietary practices and beliefs of people living with inactive ulcerative colitis. Eur. J. Gastroenterol. Hepatol..

[73] Sousa Guerreiro C., Cravo M., Costa A.R., Miranda A., Tavares L., Moura-Santos P., MarquesVidal P., Leitão C.N. (2007). A comprehensive approach to evaluate nutritional status in Crohn’s patients in the era of biologic therapy: A case-control study. Am. J. Gastroenterol..

[74] Hartman C., Marderfeld L., Davidson K., Mozer-Glassberg Y., Poraz I., Silbermintz A., Zevit N., Shamir R. (2016). Food intake adequacy in children and adolescents with inflammatory bowel disease. J. Pediatr. Gastroenterol. Nutr..

[75] Pons R., Whitten K.E., Woodhead H., Leach S.T., Lemberg D.A., Day A.S. (2009). Dietary intakes of children with Crohn’s disease. Br. J. Nutr..

[76] Miele E., Shamir R., Aloi M., Assa A., Braegger C., Bronsky J., de Ridder L., Escher J.C., Hojsak I., Kolaček S. (2018). Nutrition in paediatric inflammatory bowel disease: A Position Paper on Behalf of the Porto Inflammatory Bowel Disease Group of the European Society of Pediatric Gastroenterology, Hepatology and Nutrition. J. Pediatr. Gastroenterol. Nutr..

[77] Kuźnicki P., Neubauer K. (2021). Emerging comorbidities in inflammatory bowel disease: Eating disorders, alcohol and narcotics misuse. J. Clin. Med..

[78] Thomas A.G., Miller V., Taylor F., Maycock P., Scrimgeour C.M., Rennie M.J. (1992). Whole body protein turnover in childhood Crohn’s disease. Gut.

[79] Steiner S.J., Noe J.D., Denne S.C. (2011). Corticosteroids increase protein breakdown and loss in newly diagnosed pediatric Crohn disease. Pediatr. Res..

[80] Gerasimidis K., Edwards C., Stefanowicz F., Galloway P., McGrogan P., Duncan A., Talwar D. (2013). Micronutrient status in children with IBD. J. Pediatr. Gastroenterol. Nutr..

[81] Filippi J., Al-Jaouni R., Wiroth J.-B., Hébuterne X., Schneider S.M. (2006). Nutritional deficiencies in patients with Crohn’s disease in remission. Inflamm. Bowel Dis..

[82] Vagianos K., Bector S., McConnell J., Bernstein C.N. (2007). Nutrition assessment of patients with inflammatory bowel disease. JPEN J. Parenter. Enter. Nutr..

[83] Santucci N.R., Alkhouri R.H., Baker R.D., Baker S.S. (2014). Vitamin and zinc status pretreatment and posttreatment in patients with inflammatory bowel disease. J. Pediatr. Gastroenterol. Nutr..

[84] Sasson A.N., Ananthakrishnan A.N., Raman M. (2021). Diet in treatment of inflammatory bowel diseases. Clin. Gastroenterol. Hepatol..

[85] Di Caro S., Fragkos K.C., Keetarut K., Koo H.F., Sebepos-Rogers G., Saravanapavan H., Barragry J., Rogers J., Mehta S.J., Rahman F. (2019). Enteral Nutrition in Adult Crohn’s Disease: Toward a Paradigm Shift. Nutrients.

[86] Yamamoto T., Shimoyama T., Kuriyama M. (2017). Dietary and enteral interventions for Crohn’s disease. Curr. Opin. Biotechnol..

[87] Nakahigashi M., Yamamoto T., Sacco R., Hanai H., Kobayashi F. (2016). Enteral nutrition for maintaining remission in patients with quiescent Crohn’s disease: Current status and future perspectives. Int. J. Color. Dis..

[88] Whitten K.E., Rogers P., Ooi C.Y., Day A.S. (2012). International survey of enteral nutrition protocols used in children with Crohn’s disease. J. Dig. Dis..

[89] Ashton J.J., Gavin J., Beattie R.M. (2019). Exclusive enteral nutrition in Crohn’s disease: Evidence and practicalities. Clin. Nutr..

[90] Gerasimidis K., Bertz M., Hanske L., Junick J., Biskou O., Aguilera M., Garrick V., Russell R.K., Blaut M., McGrogan P. (2014). Decline in presumptively protective gut bacterial species and metabolites are paradoxically associated with disease improvement in pediatric Crohn’s disease during enteral nutrition. Inflamm. Bowel Dis..

[91] D’Argenio V., Precone V., Casaburi G., Miele E., Martinelli M., Staiano A., Salvatore F., Sacchetti L. (2013). An altered gut microbiome profile in a child affected by Crohn’s disease normalized after nutritional therapy. Am. J. Gastroenterol..

[92] Lewis J.D., Chen E.Z., Baldassano R.N., Otley A.R., Griffiths A.M., Lee D., Bittinger K., Bailey A., Friedman E.S., Hoffmann C. (2015). Inflammation, Antibiotics, and Diet as Environmental Stressors of the Gut Microbiome in Pediatric Crohn’s Disease. Cell Host Microbe.

[93] Guinet-Charpentier C., Lepage P., Morali A., Chamaillard M., Peyrin-Biroulet L. (2017). Effects of enteral polymeric diet on gut microbiota in children with Crohn’s disease. Gut.

[94] MacLellan A., Moore-Connors J., Grant S., Cahill L., Langille M.G.I., Van Limbergen J. (2017). The Impact of Exclusive Enteral Nutrition (EEN) on the Gut Microbiome in Crohn’s Disease: A Review. Nutrients.

[95] Pigneur B., Ruemmele F.M. (2019). Nutritional interventions for the treatment of IBD: Current evidence and controversies. Ther. Adv. Gastroenterol..

[96] Soo J., Malik B.A., Turner J.M., Persad R., Wine E., Siminoski K., Huynh H.Q. (2013). Use of exclusive enteral nutrition is just as effective as corticosteroids in newly diagnosed pediatric Crohn’s disease. Dig. Dis. Sci..

[97] Luo Y., Yu J., Zhao H., Lou J., Chen F., Peng K., Chen J. (2015). Short-Term Efficacy of Exclusive Enteral Nutrition in Pediatric Crohn’s Disease: Practice in China. Gastroenterol. Res. Pract..

[98] Verburgt C.M., Dunn K.A., Ghiboub M., Lewis J.D., Wine E., Sigall Boneh R., Gerasimidis K., Shamir R., Penny S., Pinto D.M. (2023). Successful Dietary Therapy in Paediatric Crohn’s Disease is Associated with Shifts in Bacterial Dysbiosis and Inflammatory Metabotype Towards Healthy Controls. J. Crohns Colitis.

[99] Richman E., Rhodes J.M. (2013). Review article: Evidence-based dietary advice for patients with inflammatory bowel disease. Aliment. Pharmacol. Ther..

[100] Faiman A., Mutalib M., Moylan A., Morgan N., Crespi D., Furman M., Kader A. (2014). Standard versus rapid food reintroduction after exclusive enteral nutritional therapy in paediatric Crohn’s disease. Eur. J. Gastroenterol. Hepatol..

[101] Yamamoto T., Shiraki M. (2013). Efficacy of enteral nutrition during infliximab maintenance therapy in patients with Crohn’s disease. Dig. Dis. Sci..

[102] Gavin J., Ashton J.J., Heather N., Marino L.V., Beattie R.M. (2018). Nutritional support in paediatric Crohn’s disease: Outcome at 12 months. Acta Paediatr..

[103] Werkstetter K.J., Schatz S.B., Alberer M., Filipiak-Pittroff B., Koletzko S. (2013). Influence of exclusive enteral nutrition therapy on bone density and geometry in newly diagnosed pediatric Crohn’s disease patients. Ann. Nutr. Metab..

[104] Brown S.C., Wall C.L., Gearry R.B., Day A.S. (2023). Exclusive Enteral Nutrition for the Treatment of Pediatric Crohn’s Disease: The Patient Perspective. Pediatr. Gastroenterol. Hepatol. Nutr..

[105] Cuomo M., Carobbio A., Aloi M., Alvisi P., Banzato C., Bosa L., Bramuzzo M., Campanozzi A., Catassi G., D’Antiga L. (2023). Induction of Remission with Exclusive Enteral Nutrition in Children with Crohn’s Disease: Determinants of Higher Adherence and Response. Inflamm. Bowel Dis..

[106] Cucinotta U., Romano C., Dipasquale V. (2021). Diet and Nutrition in Pediatric Inflammatory Bowel Diseases. Nutrients.

[107] Wang G., Ren J., Li G., Hu Q., Gu G., Ren H., Hong Z., Li J. (2018). The utility of food antigen test in the diagnosis of Crohn’s disease and remission maintenance after exclusive enteral nutrition. Clin. Res. Hepatol. Gastroenterol..

[108] Lee D., Baldassano R.N., Otley A.R., Albenberg L., Griffiths A.M., Compher C., Chen E.Z., Li H., Gilroy E., Nessel L. (2015). Comparative Effectiveness of Nutritional and Biological Therapy in North American Children with Active Crohn’s Disease. Inflamm. Bowel Dis..

[109] Sigall Boneh R., Park S., Soledad Arcucci M., Herrador-López M., Sarbagili-Shabat C., Kolonimos N., Wierdsma N., Chen M., Hershkovitz E., Wine E. (2024). Cultural Perspectives on the Efficacy and Adoption of the Crohn’s Disease Exclusion Diet across Diverse Ethnicities: A Case-Based Overview. Nutrients.

[110] Damas O.M., Maldonado-Contreras A. (2023). Breaking Barriers in Dietary Research: Strategies to Diversify Recruitment in Clinical Studies and Develop Culturally Tailored Diets for Hispanic Communities Living with Inflammatory Bowel Disease. Gastroenterology.

[111] Arcucci M.S., Menendez L., Orsi M., Gallo J., Guzman L., Busoni V., Lifschitz C. (2024). Role of adjuvant Crohn’s disease exclusion diet plus enteral nutrition in asymptomatic pediatric Crohn’s disease having biochemical activity: A randomized, pilot study. Indian J. Gastroenterol..

[112] Landorf E., Hammond P., Abu-Assi R., Ellison S., Boyle T., Comerford A., Couper R. (2024). Formula modifications to the Crohn’s disease exclusion diet do not impact therapy success in paediatric Crohn’s disease. J. Pediatr. Gastroenterol. Nutr..

[113] Hracs L., Windsor J.W., Gorospe J., Cummings M., Coward S., Buie M.J., Quan J., Goddard Q., Caplan L., Markovinović A. (2025). Global evolution of inflammatory bowel disease across epidemiologic stages. Nature.

[114] Epstein D., Kassianides C., Gaibee Z., Watermeyer G., Griffiths A. (2020). Inflammatory bowel disease in children in sub-Saharan Africa. Paediatr. Int. Child Health.

[115] Scarallo L., Lionetti P. (2021). Dietary Management in Pediatric Patients with Crohn’s Disease. Nutrients.

[116] Hauer A.C., Sultan M., Darma A., Altamimi E., Serban D.E., Assa A., Franco C.P.S., de Ridder L., Wilson D.C., Afzal N.A. (2025). Management of pediatric inflammatory bowel diseases in limited-resource settings: A position paper from the Paediatric IBD Porto Group of ESPGHAN. J. Pediatr. Gastroenterol. Nutr..

[117] Sigall Boneh R., Sarbagili Shabat C., Yanai H., Chermesh I., Ben Avraham S., Boaz M., Levine A. (2017). Dietary Therapy with the Crohn’s Disease Exclusion Diet is a Successful Strategy for Induction of Remission in Children and Adults Failing Biological Therapy. J. Crohns Colitis.

[118] Niseteo T., Sila S., Trivić I., Mišak Z., Kolaček S., Hojsak I. (2022). Modified Crohn’s disease exclusion diet is equally effective as exclusive enteral nutrition: Real-world data. Nutr. Clin. Pract..

[119] Matuszczyk M., Meglicka M., Wiernicka A., Jarzębicka D., Osiecki M., Kotkowicz-Szczur M., Kierkuś J. (2022). Effect of the Crohn’s Disease Exclusion Diet (CDED) on the Fecal Calprotectin Level in Children with Active Crohn’s Disease. J. Clin. Med..

[120] Jijón Andrade M.C., Pujol Muncunill G., Lozano Ruf A., Álvarez Carnero L., Vila Miravet V., García Arenas D., Egea Castillo N., Martín de Carpi J. (2023). Efficacy of Crohn’s disease exclusion diet in treatment -naïve children and children progressed on biological therapy: A retrospective chart review. BMC Gastroenterol..

[121] Cohen S.A., Gold B.D., Oliva S., Lewis J., Stallworth A., Koch B., Eshee L., Mason D. (2014). Clinical and mucosal improvement with specific carbohydrate diet in pediatric Crohn disease. J. Pediatr. Gastroenterol. Nutr..

[122] Suskind D.L., Lee D., Kim Y.M., Wahbeh G., Singh N., Braly K., Nuding M., Nicora C.D., Purvine S.O., Lipton M.S. (2020). The Specific Carbohydrate Diet and Diet Modification as Induction Therapy for Pediatric Crohn’s Disease: A Randomized Diet Controlled Trial. Nutrients.

[123] Wahbeh G.T., Ward B.T., Lee D.Y., Giefer M.J., Suskind D.L. (2017). Lack of Mucosal Healing from Modified Specific Carbohydrate Diet in Pediatric Patients with Crohn Disease. J. Pediatr. Gastroenterol. Nutr..

[124] Gibson P.R., Yao C.K., Halmos E.P. (2024). Review article: Evidence-based dietary management of inflammatory bowel disease. Aliment. Pharmacol. Ther..

[125] Braly K., Williamson N., Shaffer M.L., Lee D., Wahbeh G., Klein J., Giefer M., Suskind D.L. (2017). Nutritional Adequacy of the Specific Carbohydrate Diet in Pediatric Inflammatory Bowel Disease. J. Pediatr. Gastroenterol. Nutr..

[126] Biesiekierski J.R., Peters S.L., Newnham E.D., Rosella O., Muir J.G., Gibson P.R. (2013). No effects of gluten in patients with self-reported non-celiac gluten sensitivity after dietary reduction of fermentable, poorly absorbed, short-chain carbohydrates. Gastroenterology.

[127] Leach S.T., Mitchell H.M., Eng W.R., Zhang L., Day A.S. (2008). Sustained modulation of intestinal bacteria by exclusive enteral nutrition used to treat children with Crohn’s disease. Aliment. Pharmacol. Ther..

[128] Peng Z., Yi J., Liu X. (2022). A Low-FODMAP Diet Provides Benefits for Functional Gastrointestinal Symptoms but Not for Improving Stool Consistency and Mucosal Inflammation in IBD: A Systematic Review and Meta-Analysis. Nutrients.

[129] Strisciuglio C., Cenni S., Serra M.R., Dolce P., Martinelli M., Staiano A., Miele E. (2020). Effectiveness of Mediterranean Diet’s Adherence in children with Inflammatory Bowel Diseases. Nutrients.

[130] Merra G., Noce A., Marrone G., Cintoni M., Tarsitano M.G., Capacci A., De Lorenzo A. (2020). Influence of Mediterranean Diet on Human Gut Microbiota. Nutrients.

[131] Martínez-González M.A., Gea A., Ruiz-Canela M. (2019). The Mediterranean Diet and Cardiovascular Health. Circ. Res..

[132] Hashash J.G., Elkins J., Lewis J.D., Binion D.G. (2024). AGA Clinical Practice Update on Diet and Nutritional Therapies in Patients with Inflammatory Bowel Disease: Expert Review. Gastroenterology.

[133] Lewis J.D., Sandler R.S., Brotherton C., Brensinger C., Li H., Kappelman M.D., Daniel S.G., Bittinger K., Albenberg L., Valentine J.F. (2021). A Randomized Trial Comparing the Specific Carbohydrate Diet to a Mediterranean Diet in Adults with Crohn’s Disease. Gastroenterology.

[134] Peters V., Spooren C.E.G.M., Pierik M.J., Weersma R.K., van Dullemen H.M., Festen E.A.M., Visschedijk M.C., Masclee A.A.M., Hendrix E.M.B., Almeida R.J. (2021). Dietary Intake Pattern is Associated with Occurrence of Flares in IBD Patients. J. Crohns Colitis.

[135] Sarbagili-Shabat C., Albenberg L., Van Limbergen J., Pressman N., Otley A., Yaakov M., Wine E., Weiner D., Levine A. (2021). A Novel UC Exclusion Diet and Antibiotics for Treatment of Mild to Moderate Pediatric Ulcerative Colitis: A Prospective Open-Label Pilot Study. Nutrients.

[136] Cederholm T., Bosaeus I., Barazzoni R., Bauer J., Van Gossum A., Klek S., Muscaritoli M., Nyulasi I., Ockenga J., Schneider S. (2015). Diagnostic criteria for malnutrition—An ESPEN Consensus Statement. Clin. Nutr..

[137] Forbes A., Escher J., Hébuterne X., Kłęk S., Krznaric Z., Schneider S., Shamir R., Stardelova K., Wierdsma N., Wiskin A.E. (2017). ESPEN guideline: Clinical nutrition in inflammatory bowel disease. Clin. Nutr..

[138] Massironi S., Viganò C., Palermo A., Pirola L., Mulinacci G., Allocca M., Peyrin-Biroulet L., Danese S. (2023). Inflammation and malnutrition in inflammatory bowel disease. Lancet Gastroenterol. Hepatol..

[139] Scaldaferri F., Pizzoferrato M., Lopetuso L.R., Musca T., Ingravalle F., Sicignano L.L., Mentella M., Miggiano G., Mele M.C., Gaetani E. (2017). Nutrition and IBD: Malnutrition and/or sarcopenia? A practical guide. Gastroenterol. Res. Pract..

[140] Herzog D., Fournier N., Buehr P., Koller R., Rueger V., Heyland K., Nydegger A., Spalinger J., Schibli S., Braegger C. (2014). Early-onset Crohn’s disease is a risk factor for smaller final height. Eur. J. Gastroenterol. Hepatol..

[141] Lee J.J., Escher J.C., Shuman M.J., Forbes P.W., Delemarre L.C., Harr B.W., Kruijer M., Moret M., Allende-Richter S., Grand R.J. (2010). Final adult height of children with inflammatory bowel disease is predicted by parental height and patient minimum height Z-score. Inflamm. Bowel Dis..

[142] Walters T.D., Griffiths A.M. (2009). Mechanisms of growth impairment in pediatric Crohn’s disease. Nat. Rev. Gastroenterol. Hepatol..

[143] Sentongo T.A., Semeao E.J., Piccoli D.A., Stallings V.A., Zemel B.S. (2000). Growth, body composition, and nutritional status in children and adolescents with Crohn’s disease. J. Pediatr. Gastroenterol. Nutr..

[144] Thangarajah D., Hyde M.J., Konteti V.K.S., Santhakumaran S., Frost G., Fell J.M.E. (2015). Systematic review: Body composition in children with inflammatory bowel disease. Aliment. Pharmacol. Ther..

[145] Thayu M., Denson L.A., Shults J., Zemel B.S., Burnham J.M., Baldassano R.N., Howard K.M., Ryan A., Leonard M.B. (2010). Determinants of changes in linear growth and body composition in incident pediatric Crohn’s disease. Gastroenterology.

[146] Long M.D., Crandall W.V., Leibowitz I.H., Duffy L., del Rosario F., Kim S.C., Integlia M.J., Berman J., Grunow J., Colletti R.B. (2011). Prevalence and epidemiology of overweight and obesity in children with inflammatory bowel disease 12. Inflamm. Bowel Dis..

[147] Kugathasan S., Nebel J., Skelton J.A., Markowitz J., Keljo D., Rosh J., LeLeiko N., Mack D., Griffiths A., Bousvaros A. (2007). Body mass index in children with newly diagnosed inflammatory bowel disease: Observations from two multicenter North American inception cohorts. J. Pediatr..

[148] Singh S., Dulai P.S., Zarrinpar A., Ramamoorthy S., Sandborn W.J. (2017). Obesity in IBD: Epidemiology, pathogenesis, disease course and treatment outcomes. Nat. Rev. Gastroenterol. Hepatol..

[149] DeBoer M.D., Denson L.A. (2013). Delays in puberty, growth, and accrual of bone mineral density in pediatric Crohn’s disease: Despite temporal changes in disease severity, the need for monitoring remains. J. Pediatr..

[150] Ballinger A.B., Savage M.O., Sanderson I.R. (2003). Delayed puberty associated with inflammatory bowel disease. Pediatr. Res..

[151] Abraham B.P., Prasad P., Malaty H.M. (2014). Vitamin D deficiency and corticosteroid use are risk factors for low bone mineral density in inflammatory bowel disease patients. Dig. Dis. Sci..

[152] Bakker S.F., Dik V.K., Witte B.I., Lips P., Roos J.C., Van Bodegraven A.A. (2013). Increase in bone mineral density in strictly treated Crohn’s disease patients with concomitant calcium and vitamin D supplementation. J. Crohns Colitis.

[153] Lopes L.H.C., Sdepanian V.L., Szejnfeld V.L., de Morais M.B., Fagundes-Neto U. (2008). Risk factors for low bone mineral density in children and adolescents with inflammatory bowel disease. Dig. Dis. Sci..

[154] van Bodegraven A.A., Bravenboer N., Witte B.I., Dijkstra G., van der Woude C.J., Stokkers P.C.M., Russel M.G., Oldenburg B., Pierik M., Roos J.C. (2014). Treatment of bone loss in osteopenic patients with Crohn’s disease: A double-blind, randomised trial of oral risedronate 35 mg once weekly or placebo, concomitant with calcium and vitamin D supplementation. Gut.

[155] Dignass A.U., Gasche C., Bettenworth D., Birgegård G., Danese S., Gisbert J.P., Gomollon F., Iqbal T., Katsanos K., Koutroubakis I. (2015). European consensus on the diagnosis and management of iron deficiency and anaemia in inflammatory bowel diseases. J. Crohns Colitis.

[156] Cao Q., Huang Y.-H., Jiang M., Dai C. (2019). The prevalence and risk factors of psychological disorders, malnutrition and quality of life in IBD patients. Scand. J. Gastroenterol..

[157] Czuber-Dochan W., Morgan M., Hughes L.D., Lomer M.C.E., Lindsay J.O., Whelan K. (2020). Perceptions and psychosocial impact of food, nutrition, eating and drinking in people with inflammatory bowel disease: A qualitative investigation of food-related quality of life. J. Hum. Nutr. Diet..

[158] Gamwell K.L., Roberts C.M., Espeleta H.C., Baudino M.N., Hommel K.A., Grunow J.E., Jacobs N.J., Gillaspy S.R., Mullins L.L., Chaney J.M. (2020). Perceived stigma, illness uncertainty, and depressive symptoms in youth with inflammatory bowel disease: The moderating effect of mindfulness. Psychol. Health Med..

[159] Kaul K., Schumann S., Felder J., Däbritz J., de Laffolie J. (2025). Patient Empowerment Among Children and Adolescents with Inflammatory Bowel Disease (IBD) and Parents of IBD Patients-Use of Counseling Services and Lack of Knowledge About Transition. Children.

[160] Eindor-Abarbanel A., Pinchevski N., Shalem T., Agajany N., Ophir N., Weiss B., Broide E., Richter V. (2024). Parental perspectives on pediatric inflammatory bowel disease: Unraveling concerns, and study participation willingness. J. Pediatr. Gastroenterol. Nutr..

